# Prevalence of imperfect amelogenesis cases in a paediatric population of the paediatric dentistry clinic of IUEM

**DOI:** 10.1080/07853890.2021.1897376

**Published:** 2021-09-28

**Authors:** G. Kizi, O. Toga, A.R. Barata, A. Castaño Séiquer, I. Ventura

**Affiliations:** a Centro de Investigação Interdisciplinar Egas Moniz (CiiEM), Egas Moniz Cooperativa de Ensino Superior, Caparica, Portugal; b Instituto Universitário Egas Moniz (IUEM), Egas Moniz Cooperativa de Ensino Superior, Caparica, Caparica, Portugal; c Family and Community Dentistry, Sevilla University, Spain

## Abstract

**Introduction:**

The imperfect amelogenesis (IA) is a heterogeneous group of changes that mainly affect the structure of the enamel. It can occur in the deciduous and permanent dentition and presents a variation of phenotype, associated or not to a syndrome [1,2]. The aim of this study is to evaluate the prevalence of IA in patients who were observed for the first time in the Egas Moniz University Clinic and to describe which teeth are most affected. Variables such as sex, age, oral hygiene, diet and DMFT index (decay-missing-filling-teeth) were evaluated. DMFT index was analysed in order to determine if in the presence of enamel changes, correct oral hygiene and low sugar diet, the DMFT index remains high, to determine if the enamel changes have an impact on dental caries in the absence of external factors.

**Materials and methods:**

The study was approved by an Ethics Committee of Egas Moniz and a written consent was obtained from all participants.The sample consisted of 100 children who attended Egas Moniz paediatric dentistry, for the first time, over a two-year period (2015–2017). Inclusion criteria were: Children without craniofacial changes and that the parents have signed the informed consent. Data were analysed by using descriptive and inferential methodologies. A significance level of 5% was established in the latter case.

**Results:**

The mean age was 10.2 years, ranging from 6 to 18 years. The majority (55%) was male and 45% was female. Permanent dentition is the most prevalent (52%), followed by mixed (30%) and deciduous (18%). Of the total sample, it was observed 14% of patients with IA. The permanent dentition presents 50% of IA, followed by 42.9% in the deciduous dentition, reducing in the mixed dentition (7,10%). The most affected teeth were the second upper molars with a percentage of 13%. Regarding the relation of the changes with the gender, age, DMFT index, and distribution by quadrants, did not show statistically significant differences (*p* > .05).

**Discussion and Conclusions:**

In this study, the prevalence of IA was higher than in other studies that revealed values of 0.3%, 0.5% and 0.27% [2–4], but close to Temilola et al. found 16.1% of cases of IA in a paediatric population in Nigeria [5]. There was no statistically significant relationship between DMFT and IA in the present study (*p* ≥ .05). Other studies have demonstrated a relationship between hyponeralization of the enamel and increased risk of caries [2,3]. The high prevalence of AI found in this study may be due to the fact that the majority of patients, with suspected rare alterations of the dental tissues, are frequently sent, to the consultation of the Egas Moniz Dental Clinic by family doctors, paediatrics and other dentists. When the dentist attends these cases, he must establish the diagnosis, inform the patient, and recommend the therapeutic approach that may have a multidisciplinary involvement.
